# Acute Thrombotic Thrombocytopenic Purpura Following Hip Hemiarthroplasty: A Case Report and Literature Review

**DOI:** 10.7759/cureus.116663

**Published:** 2026-09-21

**Authors:** Alyssa L Carlson, Harshasree Seelam, Aye Thida, Sylvester Homsy, Robert Levy

**Affiliations:** 1 College of Medicine, State University of New York Downstate Health Sciences University, Brooklyn, USA; 2 Hematology and Oncology, State University of New York Downstate Health Sciences University, Brooklyn, USA

**Keywords:** adamts13, microangiopathy, thrombocytopenia, total hip arthroplasty (tha), ttp

## Abstract

Acute thrombotic thrombocytopenic purpura (TTP) is a rare, life-threatening hematologic disorder. The spontaneous development of TTP following orthopedic surgery is an uncommon occurrence, with only five documented cases. Herein, we present a case of TTP that developed after a hemiarthroplasty of the hip. As TTP is an unexpected complication following orthopedic surgery, this case highlights the critical importance of early recognition and prompt treatment to significantly improve the likelihood of survival.

## Introduction

Thrombotic thrombocytopenic purpura (TTP) is a rare microangiopathy with an incidence of 10 cases per million people [1]. It results from deficiency or dysfunction of ADAMTS13, a metalloprotease that cleaves von Willebrand factor (vWF), and approximately 75% of cases are caused by anti-ADAMTS13 polyclonal IgG autoantibodies [1,2]. A reduction in ADAMTS13 activity can lead to unusually large vWF multimers, which promote microthrombi formation, widespread platelet consumption, and extensive small-vessel occlusion [3,4]. Although the clinical presentation may vary widely, TTP has classically been characterized by a pentad of symptoms: microangiopathic hemolytic anemia (MAHA), severe thrombocytopenia, fever, neurological disorientation, and renal dysfunction [1,3,4]. Physical findings may include petechiae, purpura, or ecchymoses related to thrombocytopenia, as well as pallor or jaundice, including scleral icterus, secondary to hemolysis [1,4]. Laboratory findings may demonstrate elevated lactate dehydrogenase (LDH), decreased haptoglobin, and schistocytes on peripheral blood smear, consistent with microangiopathic hemolysis. Renal involvement may range from mild renal dysfunction to acute kidney injury.

TTP is often caused by autoimmune disorders, medications, and rarely by congenital deficiency of ADAMTS13. To date, there have only been five reported cases of acute TTP following orthopedic surgeries, including high tibial osteotomy [5,6], total knee arthroplasty [7,8], and total hip arthroplasty [9]. Here, we report the first documented case of TTP occurring after hemiarthroplasty. This case highlights the importance of identifying TTP in rare circumstances, such as in the postoperative setting of orthopedic surgery. 

## Case presentation

A 65-year-old African American female patient with a history of human immunodeficiency virus (HIV), managed with emtricitabine and having an undetectable viral load, was admitted following a mechanical fall. She had a history of cerebrovascular accident with residual right-sided hemiparesis, aphasia, and seizures. During hospitalization, she underwent hemiarthroplasty for a right femoral neck fracture.

On postoperative day (POD) 1, routine labs were notable for a low platelet count of 115 K/uL, which progressively declined to 31 K/uL by POD 4 (Table 1). There was also an associated downtrend in hemoglobin (Hb) from 11.6 g/dL to 8.3 g/dL. Initially, thrombocytopenia was attributed to postoperative changes; however, the patient’s platelet count continued to decline, reaching 11 K/uL by POD 6, with worsening anemia, Hb 6.5 gm/dL. Coagulation tests revealed normal prothrombin time and activated partial thromboplastin time, while fibrinogen level was elevated at 846 mg/dL on POD 6. Bilirubin level increased from 0.5 mg/dL on POD 4 to 1.4 mg/dL on POD 6. Haptoglobin decreased to less than 10 mg/dL on POD 6, and LDH rose significantly from 656 U/L on POD 4 to 1,705 U/L on POD 6. Reticulocyte count increased from 0.081 M/uL to 0.237 M/uL, suggesting possible hemolysis; however, direct Coombs testing was performed and returned negative. On POD 6, peripheral smear review revealed polychromasia and a marked increase in schistocytes (8-12 HPF). Her renal function remained normal. Collectively, these laboratory findings were most consistent with microangiopathic hemolysis. The calculated PLASMIC score (platelet count, hemolysis, absence of active cancer, absence of solid organ or stem cell transplant, mean corpuscular volume, international normalized ratio, and creatinine) [10] was 7 points, indicating a high risk for TTP. An ADAMTS13 level was ordered, and treatment was initiated with plasma exchange (PLEX) and oral prednisone at a dosage of 1 mg/kg daily. 

**Table 1:** Patient laboratory values POD: postoperative day; TTP: thrombotic thrombocytopenic purpura

Laboratory Study	Patient Value			Reference Range
	POD 1	POD 4	POD 6	
Platelet count (K/uL)	115	31	11	150-400 K/uL
Hemoglobin (g/dL)	11.6	8.3	-	12.0-16.0 g/dL (female)
Fibrinogen (mg/dL)	-	-	846	200-400 mg/dL
Bilirubin (mg/dL)	-	0.5	1.4	0.2-1.2 mg/dL
Haptoglobin (mg/dL)	-	-	<10	30-200 mg/dL
Lactate dehydrogenase (U/L)	-	656	1,705	140-280 U/L
Reticulocyte count (M/uL)	-	0.081	0.237	0.02-0.10 M/uL

The diagnosis of TTP was confirmed when the ADAMTS13 level returned as <2%, along with an ADAMTS13 antibody level of 34 units/mL. The patient was started on steroids and rituximab on days 6 and 7. Following seven sessions of PLEX, the patient’s platelets normalized. Clinically, the patient remained stable with no petechiae and no changes in hemodynamics or mentation. However, two days after discontinuing PLEX, LDH rose from 320 U/L to 405 U/L. As a result, PLEX was restarted for an additional two sessions. During this treatment period, further PLEX was held due to hypotension. However, due to the continued decline in haptoglobin and increased schistocytes on peripheral smear, PLEX was reinitiated five days later. The ADAMTS13 level was subsequently confirmed to be <5%, further supporting ongoing TTP. After a few more days of PLEX, hemolysis labs improved, and the ADAMTS13 level rose to 29%, with low antibody levels. The patient was eventually discharged to a skilled nursing facility. 

During her follow-up clinic visit, no petechiae were observed upon examination, and her cardiopulmonary assessment was unremarkable. The last complete blood count (CBC) revealed normal platelet count and Hb. The patient's HIV viral load was undetectable throughout, and she developed no opportunistic infections. At the three-month interval follow-up, the patient remained clinically stable with no recurrence of TTP.

## Discussion

Acute TTP is a rare phenomenon following orthopedic surgery, especially in patients without a prior history of TTP. Our case represents the first report of acute TTP following hemiarthroplasty of the hip in the literature, which was successfully treated with PLEX, prednisone, and rituximab. Notably, our patient exhibited only two of the five classic symptoms of TTP (severe thrombocytopenia and MAHA), emphasizing that postoperative presentations of TTP may deviate from the classic presentation.

Our case adds to the other five documented cases of TTP following uncomplicated orthopedic surgeries (Table 2). These cases include high tibial osteotomy [5,6], total knee arthroplasty [7,8], and total hip arthroplasty [9]. Including our case, the mean POD of onset was 3.33 days (standard deviation 2.66, n = 6), and all cases occurred in female patients. Each TTP presentation varied from the classically described pentad (MAHA, severe thrombocytopenia, fever, confusion, and renal dysfunction) (Table 2). All six patients shared only MAHA and thrombocytopenia in common. Additionally, three of the five cases reported nausea and vomiting, which are not typical symptoms associated with TTP [5,6,9]. Given that TTP is already a rare and unexpected complication after surgery, these cases underscore the critical need for physicians to include TTP as a possible differential diagnosis when patients exhibit unexplained, significant decreases in platelet count and Hb levels in the acute postoperative setting.

**Table 2:** Reported cases of TTP following orthopedic surgery MAHA, microangiopathic hemolytic anemia; PLEX, plasma exchange; POD, postoperative day; TTP, thrombotic thrombocytopenic purpura; F, female

Author	Age, Sex	Past Medical/Surgical History	Type of Orthopedic Surgery	Perioperative Medications	POD to TTP Onset	Clinical Presentation	Pentad Presentation	Treatment	Outcome
Yenigun et al. [5]	15, F	Cerebral palsy, genu valgum	High tibial valgus osteotomy	Ampicillin-sulbactam	2	Nausea, vomiting, oliguria, lower extremity edema	3/5 (thrombocytopenia, MAHA, renal dysfunction)	PLEX (n = 14), prednisone, aspirin	Recovered
Kathula et al. [7]	52, F	Hypertension, cholecystectomy, hysterectomy, carpal tunnel syndrome	Total knee arthroplasty	Ticlopidine, atenolol, lorazepam, diphenhydramine; low-molecular-weight heparin (postoperatively)	1	Fever, continuous bleeding from surgical site	4/5 (thrombocytopenia, MAHA, renal failure, fever)	PLEX (n = 4)	Recovered
Shim et al. [8]	80, F	Hypertension	Total knee arthroplasty	Not reported	1	Altered mental status, right-sided heart failure	4/5 (thrombocytopenia, MAHA, renal dysfunction, altered mental status)	PLEX (n = 4)	Recovered
Patel et al. [9]	57, F	Hypertension, asthma, hip osteoarthritis	Total hip arthroplasty	Not reported	8	Fatigue, shortness of breath, nausea, vomiting, altered mental status	4/5 (thrombocytopenia, MAHA, renal dysfunction, altered mental status)	PLEX (n = 6), corticosteroids, red blood cell transfusions (n = 3)	Recovered
Iosifidis et al. [6]	57, F	Knee osteoarthritis	High tibial osteotomy	Second-generation cephalosporin	4	Fever, dizziness, nausea, vomiting, hematuria, altered mental status	5/5 (thrombocytopenia, MAHA, renal dysfunction, fever, altered mental status)	PLEX (n = 7), prednisolone, dipyridamole, aspirin, γ-globulin	Recovered

Delays in the diagnosis and management of TTP can lead to serious complications, including kidney failure, stroke, or heart failure [4,11,12]. Untreated acute TTP has a mortality rate of more than 90% [11], and even with appropriate management, mortality remains 10-20% [1,12]. Therapeutic PLEX, with or without steroids, is the primary and most effective treatment for acute TTP regardless of the cause/trigger [12,13]. Typically, patients recover fully following once- or twice-daily PLEX until platelet count rises to >150 x 10^9^/L and other end-organ symptoms resolve [13]. This process is thought to replenish ADAMTS13 levels while reducing vWF multimers, anti-ADAMTS13 autoantibodies, or other possible inhibitors from the blood [13]. In the six cases reviewed here, all patients experienced full recovery after multiple PLEX treatments with or without steroids, highlighting the efficacy of this treatment in orthopedic-surgery-related cases of TTP (Tables 2-3). 

**Table 3:** Key abnormal laboratory findings reported in cases of postoperative TTP following orthopedic surgery PLT, platelet count; Hb, hemoglobin; LDH, lactate dehydrogenase; sCr, serum creatinine; BUN, blood urea nitrogen; CK-MB, creatine kinase-myocardial band; AST, aspartate aminotransferase; ALT, alanine aminotransferase; ALP, alkaline phosphatase; GGT, gamma-glutamyl transferase; NR, not reported.

Author	PLT (×10³/mm³)	Hb (g/dL)	LDH (U/L or IU/L)	sCr (mg/dL)	BUN (mg/dL)	Total Bilirubin (mg/dL)	Other Abnormal Findings	Reference Values
Yenigun et al. [5]	23	6.6	1,280 IU/L	4.74	NR	NR	Urine protein 2,700 mg/day; schistocytes present	PLT: 150-450 ×10³/mm³; Hb: 12-16 g/dL (female); LDH: 140-280 U/L; sCr: 0.6-1.2 mg/dL; BUN: 7-20 mg/dL; total bilirubin: 0.2-1.2 mg/dL; urine protein: <150 mg/day; troponin-I: <0.04 µg/mL; CK-MB: <5 ng/mL; ADAMTS13 activity: ~50-150%; AST: 10-40 U/L; ALT: 7-56 U/L; ALP: 44-147 U/L; GGT: 9-48 U/L
Kathula et al. [7]	69	10.1	1,026 U/L	3	33	2.1	Schistocytes present	
Shim et al. [8]	20	8.6	447 IU/L	2	34	2.1	Troponin-I 0.637 µg/mL; CK-MB 8.7 ng/mL; schistocytes present	
Patel et al. [9]	6	5.4	1,189 U/L	NR	NR	NR	ADAMTS13 activity <3%; ADAMTS13 inhibitor positive; schistocytes present	
Iosifidis et al. [6]	28.9	6.41	3,062 U/L	1.3	86	1.7	AST 254 U/L; ALT 111 U/L; ALP 96 U/L; GGT 74 U/L; schistocytes present	

Our case is the first among the orthopedic-related reports to utilize rituximab in the clinical treatment course. Rituximab is a humanized monoclonal antibody targeting CD20 on B-lymphocytes, commonly utilized in the management of various malignancies and autoimmune disorders. Over the past 20 years, it has also been used in the treatment of immune-mediated TTP [14]. In cases of acute TTP episodes, the initial administration of rituximab alongside PLEX and corticosteroids has been shown to achieve remission in over 90% of patients within a span of 14 to 21 days [15]. Additionally, rituximab may help reduce the incidence of future relapses [15]. Our patient required multiple sessions of PLEX due to recurrent decreases in haptoglobin and platelet counts, along with immunosuppressive treatment using rituximab and prednisone. Ultimately, she successfully achieved remission with these treatments.

The etiology of TTP remains elusive, though certain associations have been identified. Notably, TTP is twice as common in women, and additional risk factors include Black ethnicity and the HLA-DRB1*11 allele [1,4,16]. Acute TTP has been most frequently observed following certain precipitating events such as infections, systemic lupus erythematosus (SLE), pregnancy, cancer, and organ transplantation [1,4]. While less commonly associated with surgery, TTP has been described following cardiac, vascular, abdominal, and orthopedic surgeries [5-9,17,18].

The pathophysiological mechanisms underlying the spontaneous onset of TTP after surgery are largely unknown, and hypotheses are centered around the stresses involved, such as endothelial damage, disturbances in blood flow, inflammation, and pharmacological influences [1,4,19,20]. Inflammation may directly influence platelet adhesion and vWF cleavage, as evidence suggests that hypochlorous acid, produced by inflammatory neutrophils, can deactivate ADAMTS13 and impede vWF cleavage [19]. 

Certain medications have been implicated in acute TTP. Authors of two orthopedic-related case reports consider the role of pharmaceuticals. Kathula et al. [7] speculate that recent ticlopidine use may have been a contributing factor in their case of TTP following total knee arthroplasty. Ticlopidine has been shown to have the strongest association with TTP and induces the formation of anti-ADAMTS13 autoantibodies in one in 5000 patients [20]. Iosifidis et al. [6] suggest that the prophylactic β-lactam antibiotic cephalosporin may be associated with their case of TTP following high tibial osteotomy. Other drugs have also been implicated, including clopidogrel, cyclosporine, quinine, and mitomycin-C [1,2,4]. Further research is needed to uncover the precipitating causes of de novo TTP as well as the mechanisms underlying these associations. 

## Conclusions

The sudden onset of acute TTP following orthopedic surgery underscores the critical importance of recognizing TTP as a potential post-surgical complication. Timely intervention is urgently needed in these cases, and vigilant monitoring of signs of TTP, such as unexplained MAHA and severe thrombocytopenia, is necessary in the immediate postoperative period. These cases further emphasize the need for additional research into the associations, predispositions, and etiology of acute TTP.
